# Therapeutic Potential of Sodium-Glucose Cotransporter 2 (SGLT2) Inhibitors in Liver Disease: Focus on Cirrhosis

**DOI:** 10.7759/cureus.84768

**Published:** 2025-05-25

**Authors:** Hatem Ahmed, Sameh Gomaa, Eyad Abdulrazzak, Imad Alabdul Razzak, Kellen K Kovalovich

**Affiliations:** 1 Internal Medicine, Phoenixville Hospital, Tower Health Medical Group, Phoenixville, USA; 2 Medicine, Beth Israel Deaconess Medical Center, Boston, USA; 3 Gastroenterology and Hepatology, Gastroenterology Group of Penn Specialty Practices, Limerick, USA

**Keywords:** anti-inflammatory effect, compensated liver cirrhosis disease, complications of cirrhosis, decompensated liver cirrhosis, diuretic-resistant ascites, hepatocellular carcinoma (hcc), hepatorenal syndrome (hrs), liver cirrhosis, sglt2-inhibitors, sodium-glucose cotransporter-2 (sglt2) inhibitors

## Abstract

Cirrhosis, a progressive condition characterized by hepatic fibrosis and functional decline, remains a significant global health burden. Despite advancements in understanding its pathophysiology, effective therapies to halt or reverse progression are limited, especially in advanced stages. Cirrhosis decompensation represents an inflection point in the disease course, with a substantial increase in mortality. Ascites is the most common decompensating event, associated with significant morbidity and healthcare burden, making it a key target in the management of decompensated cirrhosis.

Sodium-glucose cotransporter 2 (SGLT2) inhibitors, widely used for type 2 diabetes mellitus, have demonstrated potential beyond glucose regulation. Evidence from clinical and preclinical studies suggests that SGLT2 inhibitors may improve hepatic parameters, reduce hepatic steatosis and fibrosis, and mitigate complications such as ascites.

This review explores the multifaceted effects of SGLT2 inhibitors on cirrhosis, focusing on their mechanisms, clinical implications, and therapeutic potential in cirrhosis. By addressing current gaps in therapeutic strategies, SGLT2 inhibitors may represent a novel avenue for improving outcomes in patients with cirrhosis.

## Introduction and background

Liver cirrhosis remains a global health challenge with significant morbidity and mortality [1]. Chronic liver damage from diverse etiologies culminates in fibrosis and cirrhosis, which is typically characterized by complications that profoundly impact quality of life [1]. Recent advancements in understanding cirrhosis have introduced the concept of recompensation, a stage where effective management and resolution of decompensating events can stabilize liver function and improve survival outcomes [2].

Sodium-glucose cotransporter 2 (SGLT2) inhibitors, initially developed as antidiabetic medications, have gained attention for their pleiotropic effects, including potential benefits in liver disease. This review highlights the emerging role of SGLT2 inhibitors in cirrhosis and their potential to mitigate disease‑related complications.

A focused narrative review was conducted in accordance with the Preferred Reporting Items for Systematic Reviews and Meta-Analyses (PRISMA) guidelines for scoping reviews. PubMed and Google Scholar were systematically searched from database inception through December 2024 using combinations of the following terms: “sodium‑glucose cotransporter‑2 inhibitors”, “SGLT2 inhibitors”, “empagliflozin”, “dapagliflozin”, “canagliflozin”, “ertugliflozin”, “liver cirrhosis”, “decompensated cirrhosis”, and “portal hypertension”. Additional keywords, including “hepatorenal syndrome”, “ascites”, “variceal bleeding”, “inflammation”, and “hepatocellular carcinoma”, were applied to capture cirrhosis‑specific complications. Eligible publications included randomized controlled trials, observational studies, meta‑analyses, and relevant animal or mechanistic reports that evaluated the effects of SGLT2 inhibitors.

Background on cirrhosis

Cirrhosis is a progressive condition marked by diffuse hepatic fibrosis, resulting from chronic liver damage and inflammation caused by various underlying conditions. The most common etiologies include metabolic dysfunction-associated steatohepatitis (MASH), viral hepatitis, alcohol-related liver disease, and autoimmune hepatitis. Clinically, cirrhosis progresses from an asymptomatic compensated stage to decompensated cirrhosis, which is characterized by complications such as ascites, variceal bleeding, and hepatic encephalopathy [1,3].

Cirrhosis represents a significant global and national health burden. In 2019, it accounted for 1.43 million deaths worldwide, with mortality rates continuing to rise. In the United States, approximately 2.2 million adults were living with cirrhosis as of 2023, and age-adjusted mortality has increased by nearly 50% over the past decade [3]. With the growing prevalence of alcohol-related liver disease and MASH [1], there is a need for novel preventive and therapeutic strategies.

Diagnosis typically begins with a suggestive history and physical examination, supported by laboratory tests and imaging findings. Although liver biopsy remains the diagnostic gold standard, it has largely been replaced by noninvasive modalities. Commonly, a sequential evaluation approach using nonproprietary scores (e.g., fibrosis-4 index (FIB-4)), followed by elastography, offers high diagnostic performance [4]. Management focuses on treating the underlying liver disease, controlling symptoms, and managing clinical complications [3].

Recompensated cirrhosis is a recently recognized stage of liver disease, defined by the Baveno VII criteria as follows: (i) resolution of the primary liver disease etiology; (ii) disappearance of signs of decompensation (ascites, encephalopathy, and portal hypertensive bleeding) without ongoing therapy; and (iii) stable improvement in liver function tests (bilirubin, international normalized ratio, and albumin). Achieving recompensation is associated with significant survival benefits [2].

Overview of SGLT2 inhibitors

In normoglycemic individuals with normal renal function, the kidneys filter approximately 160-180 g of glucose daily, with over 99% of this glucose reabsorbed by the renal tubular system. This function is primarily mediated by SGLT2 in the proximal tubular cells [5].

SGLT2 inhibitors were introduced in 2012 for the treatment of type 2 diabetes mellitus (T2DM). Canagliflozin was the first Food and Drug Administration (FDA)-approved drug in this class, followed by dapagliflozin, empagliflozin, and ertugliflozin [6]. These insulin-independent antihyperglycemic agents are recognized for their added benefits beyond glycemic control, including improvements in renal and cardiovascular outcomes in patients with or without diabetes [7].

SGLT2 inhibitors induce sustained urinary glucose excretion, ranging from 40-80 g/day, resulting in a reduction in blood glucose levels, weight loss of approximately 2-3 kg, and enhanced lipid metabolism via hepatic gluconeogenesis and a shift in energy substrate utilization from carbohydrates to lipids [5]. Their diuretic effect, stemming from glucosuria and natriuresis, contributes to a reduction in plasma volume and blood pressure [7].

SGLT2 inhibition suppresses glucose and salt reabsorption, offering renal protective effects such as afferent arteriolar vasoconstriction and reduced intraglomerular pressure. Additionally, SGLT2 inhibitors reduce albuminuria, enhance erythropoietin release, elevate hemoglobin levels, and attenuate inflammatory markers such as interleukin-6 (IL-6), tumor necrosis factor (TNF), interferon-gamma (IFNγ), nuclear factor kappa B (NF-κβ), toll-like receptor 4 (TLR-4), and transforming growth factor-beta (TGF-β) [7].

The most common side effects of SGLT2 inhibitors include genital and urinary tract infections due to glucosuria, dehydration in elderly patients [7], and an increased risk of euglycemic diabetic ketoacidosis (DKA) [8].

Although SGLT2 is primarily expressed in the kidney, it is also present in extra-renal tissues, including the liver, heart, spleen, and brain, with the liver showing the second highest expression [9]. In hepatocytes, SGLT2 mediates glucose uptake and plays a role in fatty acid metabolism [10].

## Review

Effect on hepatic parameters

SGLT2 inhibitors have demonstrated hepatic benefits, particularly in patients with T2DM and MASH, as several studies show that these agents significantly reduce alanine aminotransferase (ALT), aspartate aminotransferase (AST), and gamma‑glutamyl transferase (GGT) levels, alongside reductions in hepatic fat content [11-15].

However, data on their effect on bilirubin levels remain conflicting. Two meta-analyses reported a significant increase in total bilirubin [14,16], whereas another showed a reduction [17]. The impact on serum albumin is similarly debated. Some studies found no significant changes [16], while others observed a modest increase of 0.8 g/L after six months of therapy [18].

SGLT2 inhibitors have also shown promise in improving liver fibrosis markers. The fibrosis-4 (FIB-4) index significantly improved after 48 weeks of treatment in patients with T2DM and metabolic dysfunction-associated steatotic liver disease (MASLD), with sustained antifibrotic effects noted over time [19]. Recent studies further confirmed reductions in hepatic steatosis and fibrosis based on biomarkers such as controlled attenuation parameter (CAP), liver stiffness measurement (LSM), and the FIB-4 index in patients who received SGLT2 inhibitors [20,21].

In a histologic evaluation, another randomized controlled trial (RCT) in patients with biopsy-proven MASLD and T2DM found that tofogliflozin was associated with a trend toward greater improvement in steatosis, ballooning, inflammation, and fibrosis compared to glimepiride over 48 weeks [22]. Notably, SGLT2 inhibitors may benefit non-diabetic patients as well. One RCT showed that empagliflozin improved liver steatosis and reduced LSM in MASLD patients without diabetes [13].

A large systematic review involving over one million patients reported that, compared to other glucose-lowering agents, SGLT2 inhibitors were associated with the most significant reductions in MASLD incidence, progression to cirrhosis, and composite liver-related events [23].

These findings suggest that SGLT2 inhibitors may provide therapeutic benefits in MASLD and potentially other forms of steatotic liver disease through both metabolic and yet-to-be-elucidated mechanisms.

Impact on sodium balance

Hyponatremia is a common finding in patients with cirrhosis, and its severity holds significant prognostic value. SGLT2 inhibitors have demonstrated the potential to improve hyponatremia through mechanisms that involve osmotic diuresis [24]. This diuretic effect preferentially eliminates free water, with proportionally less sodium being excreted. Consequently, serum sodium levels do not decline and may instead increase despite ongoing sodium excretion [25,26].

In addition to these effects, SGLT2 inhibitors have been shown to decrease atrial natriuretic peptide (ANP) levels in the blood [27]. ANP is a natriuretic peptide secreted by the atrial myocardium in response to increased atrial wall stretch due to elevated cardiac pressures. A reduction in ANP levels consequently diminishes sodium excretion and mitigates hyponatremia in patients receiving SGLT2 inhibitor therapy [28].

Furthermore, SGLT2 inhibitors inhibit sodium reabsorption in the proximal tubule, increasing sodium delivery to the macula densa downstream. This enhanced sodium delivery suppresses renin secretion and attenuates renin-angiotensin-aldosterone system (RAAS) activity. The resulting modulation of RAAS may contribute not only to the mild blood pressure reduction associated with SGLT2 inhibitor treatment but also to decreased ascites accumulation. This is particularly relevant as RAAS overactivation is a key driver of fluid retention and progression of portal hypertension in cirrhosis [29].

Effects on platelets and coagulation

The effects of SGLT2 inhibitors on platelet function and coagulation parameters remain inadequately characterized. In cirrhosis, thrombocytopenia is primarily driven by portal hypertension-induced hypersplenism and reduced thrombopoietin (TPO) production due to impaired hepatic synthesis, although direct evidence in this context is limited [30].

SGLT2 inhibitors may exert favorable effects on portal pressure through mechanisms such as osmotic diuresis and inhibition of the sympathetic nervous system [7], factors that may indirectly influence platelet counts, though this hypothesis requires further study.

Preclinical studies suggest that SGLT2 inhibitors can reduce thrombin generation and platelet granule secretion without disrupting overall hemostasis [31]. Additionally, a retrospective study comparing SGLT2 inhibitors to dipeptidyl peptidase-4 inhibitors (DPP4i) found no significant differences in platelet counts over a 12-month treatment period [15].

Further research is needed to clarify the role of SGLT2 inhibitors in cirrhosis-associated coagulopathy, particularly regarding their potential influence on platelet dynamics and bleeding risk.

Effect on inflammatory markers

Systemic inflammation plays a pivotal role in the progression of liver cirrhosis. A recent review emphasized that systemic inflammation is a key driver of acute decompensating events [32], contributing to the transition from compensated to decompensated cirrhosis [33]. Bothou et al. identified serum IL-6 as an independent predictor of decompensation in cirrhotic outpatients [34]. Trebicka et al. demonstrated that while systemic inflammation is present in all patients with acute decompensation (AD), those who progress to acute-on-chronic liver failure (ACLF) exhibit more pronounced and sustained inflammation, characterized by elevated IL-6, interleukin-8 (IL-8), interleukin-1 receptor antagonist, and human non-mercaptalbumin 2 [35].

Increased IL-6 levels have also been implicated in the development of hepatocellular carcinoma (HCC), as observed in both clinical and preclinical studies [36,37]. Similarly, tumor necrosis factor-alpha (TNF-α), through the activation of nuclear factor-kappa B and Jun N-terminal kinase, has been shown to promote HCC in mouse models [38]. Additionally, a recent study reported a strong association between inflammatory markers such as leukemia inhibitory factor, IL-6, and IL-8 and 180-day mortality in patients newly diagnosed with cirrhosis [39].

SGLT2 inhibitors have consistently demonstrated beneficial effects on inflammatory and oxidative stress markers. Studies have reported reductions in markers such as C-reactive protein (CRP), interleukin-1 (IL-1), IL-6, TNF-α, and oxidative stress biomarkers such as 8-iso-prostaglandin F2α (8-iso-PGF2α) and 8-hydroxy-2'-deoxyguanosine (8-OHdG). [40-44]. These findings suggest that SGLT2 inhibitors may exert anti-inflammatory effects beyond their glucose-lowering properties.

These anti-inflammatory effects are presumed to contribute to the cardiac and renal protective benefits of SGLT2 inhibitors [45-48]. However, despite this evidence, further research is needed to establish the direct effects of SGLT2 inhibitors on inflammatory markers, specifically in patients with liver cirrhosis. Robust studies in this population are essential to confirm these potential benefits and to elucidate the underlying mechanisms.

Effect of SGLT2 inhibitors on clinical outcomes

Ascites

Ascites, the pathological accumulation of fluid in the peritoneal cavity, is a hallmark of decompensated cirrhosis. It has a prevalence of 10% in cirrhosis patients, with nearly 60% of those with compensated cirrhosis developing ascites over a 10-year period. The condition is associated with a high three-year mortality rate of up to 50% [1].

The pathophysiology of ascites involves splanchnic vasodilation, decreased effective circulating volume, and activation of the RAAS, leading to fluid retention. Treatment typically involves diuretics, paracentesis, and advanced interventions like cell-free and concentrated ascites reinfusion therapy (CART) for refractory cases [49].

Case reports have documented the beneficial effects of SGLT2 inhibitors in patients with refractory ascites resistant to standard diuretic therapy [50-52]. These benefits are not solely attributable to their diuretic effect; SGLT2 inhibitors may improve liver function and nutritional status, increasing serum albumin levels and contributing to fluid balance [25].

A double-blinded RCT comparing dapagliflozin (10 mg/day) to standard medical therapy in patients with recurrent ascites demonstrated significantly better ascites control with dapagliflozin. However, the study also reported increased incidences of acute kidney injury and infections [53].

The mechanism of SGLT2 inhibitors suggests they may synergize with loop diuretics, potentially mitigating the “braking phenomenon” associated with prolonged loop diuretic use [54]. This synergistic action could enhance natriuresis and diuresis while preserving renal function.

Hepatic Encephalopathy

Hepatic encephalopathy (HE) is a neuropsychiatric complication of liver disease caused by the accumulation of neurotoxic substances, particularly ammonia, in the bloodstream [55]. To date, there is no evidence linking SGLT2 inhibitors to the incidence of HE in human studies.

However, preclinical data from a study investigating the effects of empagliflozin in biliary cirrhotic rats showed that SGLT2 inhibition exacerbated HE [56].

Esophageal Varices

Elevated portal hypertension is a key driver of variceal development, which is observed in up to two-thirds of patients with liver cirrhosis. Esophageal variceal bleeding, a common and severe complication of cirrhosis, is associated with a high mortality rate of 15-25% within six weeks [1].

Only one study to date has examined the relationship between SGLT2 inhibitors and esophageal varices. This large multicenter retrospective study reported that SGLT2 inhibitors significantly reduced the incidence of esophageal varices compared to DPP4i [15].

Hepatorenal Syndrome

Hepatorenal syndrome (HRS) is a severe complication of advanced cirrhosis, characterized by functional renal failure resulting from splanchnic vasodilation and reduced renal perfusion [57]. SGLT2 inhibitors may help counteract these effects by lowering intraglomerular pressure, reducing albuminuria, and exerting anti-inflammatory and antifibrotic properties [58,59].

Despite current management strategies, the prognosis for HRS remains poor, with hospital mortality rates reaching 32%, and an incidence as high as 32% among patients with advanced liver disease [60].

Limited but emerging evidence suggests that SGLT2 inhibitors may reduce the incidence of HRS. A retrospective study with a five-year follow-up demonstrated a significantly lower incidence of HRS in patients treated with SGLT2 inhibitors compared to those who were not [61]. Additionally, a randomized, double-blind study in patients with type 2 diabetes showed that dapagliflozin reduced intraglomerular pressure and post-glomerular resistance, effects that may be beneficial in the context of HRS [62].

Further large-scale studies are warranted to validate these findings and clarify the potential role of SGLT2 inhibitors in the management of HRS.

Hepatocellular Carcinoma

Cirrhosis is a precancerous condition that predisposes individuals to HCC, the most common primary liver cancer. The annual incidence of HCC in cirrhotic patients has been reported at 1.82% in a study involving two US prospective multiethnic cohorts [63]. HCC development is intricately linked to metabolic dysregulation, inflammation, and oxidative stress, areas where SGLT2 inhibitors might exert beneficial effects.

SGLT2 expression has been identified in the mitochondria of Hep3B liver cancer cells, where it regulates metabolic reprogramming and cell proliferation [10]. Cancer cells depend on glucose utilization for energy production and nucleotide synthesis. SGLT2 inhibitors selectively inhibit glucose uptake by SGLT2-expressing human liver cancer cells, such as Huh7 and HepG2 cells, reducing intracellular adenosine triphosphate (ATP) levels, inducing apoptosis, and indirectly suppressing tumor angiogenesis [64]. Furthermore, several preclinical studies have demonstrated that SGLT2 inhibitors exert direct anti-tumor effects by disrupting glucose uptake and lowering ATP production in various cancer cell types [65-67].

Animal studies further highlight the potential of SGLT2 inhibitors to modulate hallmarks of HCC, including inflammation, proliferation, and oxidative stress [68]. Clinical evidence supports these findings; for example, SGLT2 inhibitor use was associated with a lower risk of incident HCC in patients with coexisting T2DM and chronic hepatitis B infection [69]. Additionally, a meta-analysis of clinical trials revealed that SGLT2 inhibitor therapy is associated with reduced mortality in patients with HCC and T2DM [70].

Despite these promising findings, additional clinical trials are needed to confirm the utility of SGLT2 inhibitors as an adjunct therapy for HCC, especially in non-diabetic populations.

Limitations and future directions

While the emerging evidence highlights the potential benefits of SGLT2 inhibitors in liver cirrhosis, several limitations exist. Most clinical studies have focused on patients with T2DM, and evidence for non-diabetic populations remains sparse. Additionally, direct evidence supporting the use of SGLT2 inhibitors in managing complications of advanced liver disease is limited.

Ongoing clinical trials, including NCT05147090, NCT05254626, and NCT05013502, will provide critical insights into the safety and efficacy of SGLT2 inhibitors in patients with cirrhosis [71-73]. Future research should prioritize RCTs to validate the therapeutic potential of SGLT2 inhibitors in liver cirrhosis and its complications.

## Conclusions

SGLT2 inhibitors, initially developed as antidiabetic agents, have demonstrated promising effects in managing liver disease, including MASLD and cirrhosis. Their multifaceted mechanisms, including anti-inflammatory and diuretic effects, position them as potential therapeutic agents for liver disease beyond glycemic control. While preclinical and clinical evidence highlights the potential benefits of SGLT2 inhibitors in cirrhosis-related complications such as ascites, HCC, and possibly HRS, caution is necessary due to limited data and the risk of adverse effects, such as acute kidney injury and infections. Comprehensive RCTs are essential to establish their safety and efficacy in this context.

The therapeutic horizon of SGLT2 inhibitors in liver disease appears promising, and ongoing research will undoubtedly shed further light on their role in addressing the unmet needs of patients with advanced liver disease.
